# Mitophagy in the aging nervous system

**DOI:** 10.3389/fcell.2022.978142

**Published:** 2022-10-11

**Authors:** Anna Rappe, Thomas G. McWilliams

**Affiliations:** ^1^ Translational Stem Cell Biology and Metabolism Program, Research Programs Unit, Faculty of Medicine, University of Helsinki, Helsinki, Finland; ^2^ Department of Anatomy, Faculty of Medicine, University of Helsinki, Helsinki, Finland

**Keywords:** mitophagy, aging, longevity, autophagy, mitochondria, disease, brain

## Abstract

Aging is characterised by the progressive accumulation of cellular dysfunction, stress, and inflammation. A large body of evidence implicates mitochondrial dysfunction as a cause or consequence of age-related diseases including metabolic disorders, neuropathies, various forms of cancer and neurodegenerative diseases. Because neurons have high metabolic demands and cannot divide, they are especially vulnerable to mitochondrial dysfunction which promotes cell dysfunction and cytotoxicity. Mitophagy neutralises mitochondrial dysfunction, providing an adaptive quality control strategy that sustains metabolic homeostasis. Mitophagy has been extensively studied as an inducible stress response in cultured cells and short-lived model organisms. In contrast, our understanding of physiological mitophagy in mammalian aging remains extremely limited, particularly in the nervous system. The recent profiling of mitophagy reporter mice has revealed variegated vistas of steady-state mitochondrial destruction across different tissues. The discovery of patients with congenital autophagy deficiency provokes further intrigue into the mechanisms that underpin neural integrity. These dimensions have considerable implications for targeting mitophagy and other degradative pathways in age-related neurological disease.

## Introduction: Live long and prosper–why mitochondria matter for the aging brain

The human brain is notoriously energy demanding, accounting for an estimated 20% of total metabolic consumption (Engl and Attwell 2015). Mammalian neurons are long-lived post-mitotic cells that are morphologically expansive and sensitive to alterations in energy homeostasis (Chen and Chan, 2009; Engl and Attwell 2015). The mitochondrial network holds a cardinal role in eukaryotic energy metabolism and metabolic homeostasis. Yet, the textbook view of mitochondria as mere isolated ATP-producing powerhouses is outdated. Mitochondria have emerged as dynamic organelles that form a highly plastic and pleiotropic intracellular network which influences almost every aspect of cellular function (Spinelli and Haigis 2018; Giacomello et al., 2020; Tábara et al., 2021). Aside from their classical role in burning substrates and energy homeostasis (catabolism), mitochondria play major roles in building (anabolism) (Suomi and McWilliams 2019; Chakrabarty and Chandel 2021; Chandel 2021). As *bona fide* signalling organelles, the mitochondrial network contributes to iron homeostasis, amino acid, nucleotide and lipid biosynthesis, calcium handling, immune signalling, epigenetics and organelle biogenesis (Prudent and McBride, 2017; Suomi and McWilliams 2019). Accordingly, mitochondrial impairments compromise cell and tissue function, arising from a constellation of consequences including destabilising levels of reactive oxygen species, the release of pro-apoptotic factors and inflammation (Bratic and Larsson 2013).

In agreement with this, mitochondrial dysfunction is a hallmark of numerous human diseases, particularly age-related neurological disorders. Aging is described as the progressive accumulation of molecular and cellular defects that culminate in cellular dysfunction, demise and eventual death (Bratic and Larsson 2013; López-Otín et al., 2013; Cagan et al., 2022). Aging is the leading risk factor for several human neurodegenerative diseases including Alzheimer’s and Parkinson’s disease, which are a leading cause of disability and morbidity (Feigin et al., 2020). Aging is also associated with the manifestation of cancer and cardiovascular disease. This realisation creates an inevitable tension: although living longer represents a triumph of modern healthcare, our inability to reduce disease prevalence constitutes a significant challenge for healthcare systems and societies worldwide (Olesen et al., 2012). In plain English: living longer comes at a cost. Current health economics forecasts predict that dementia will affect one in three individuals in the next decade (Feigin et al., 2020). The recent coronavirus pandemic is also cause for concern, as large-scale studies demonstrate that early strains of SARS-CoV-2 have potent neuropsychiatric effects (Borah et al., 2021; Hampshire et al., 2021; Jansen van Vuren et al., 2021; Taquet et al., 2021), with neuroanatomical alterations reported in some individuals (Douaud et al., 2022). At present, it is unclear if the COVID-19 pandemic will alter or affect the prevalence of age-related neuropathology. Emerging data suggests an association between long COVID/PASC (post-acute sequalae of SARS-CoV-2) and impaired memory function and cognitive performance (Guo et al., 2022). Whether or not SARS-CoV-2 infection confers increased vulnerability to neurodegeneration and cognitive decline remains to be determined. Regardless, we urgently need to understand the protective mechanisms that sustain neural integrity throughout decades of mammalian life.

Indeed, it is possible to have a healthy nervous system throughout the aging process, as natural aging does not always result in cognitive decline and neuropathology (Young et al., 2009). Related to this, the cell biology of aging is a domain of outstanding clinical interest and understanding healthspan provokes considerable societal intrigue. The unprecedented development of sophisticated tools and experimental approaches to manipulate brain and tissue function have inspired the notion that under defined circumstances, it may be possible to delay or even reverse certain molecular hallmarks of aging (Bonkowski and Sinclair, 2016; Noroozi et al., 2021; Johnson et al., 2022).

The autophagic destruction of cellular matter, and the targeted elimination of mitochondria by selective autophagy (mitophagy) are disease relevant processes, thought to confer resilience throughout the aging process (Palikaras et al., 2015; Killackey et al., 2020; Montava-Garriga and Ganley 2020). Although we understand mitophagy at high mechanistic resolution in cultured proliferating cells, clarity on its physiological regulation in mammals is only beginning to emerge.

## Autophagy degrades cellular components to building blocks that sustain tissue health

**FIGURE 1:**
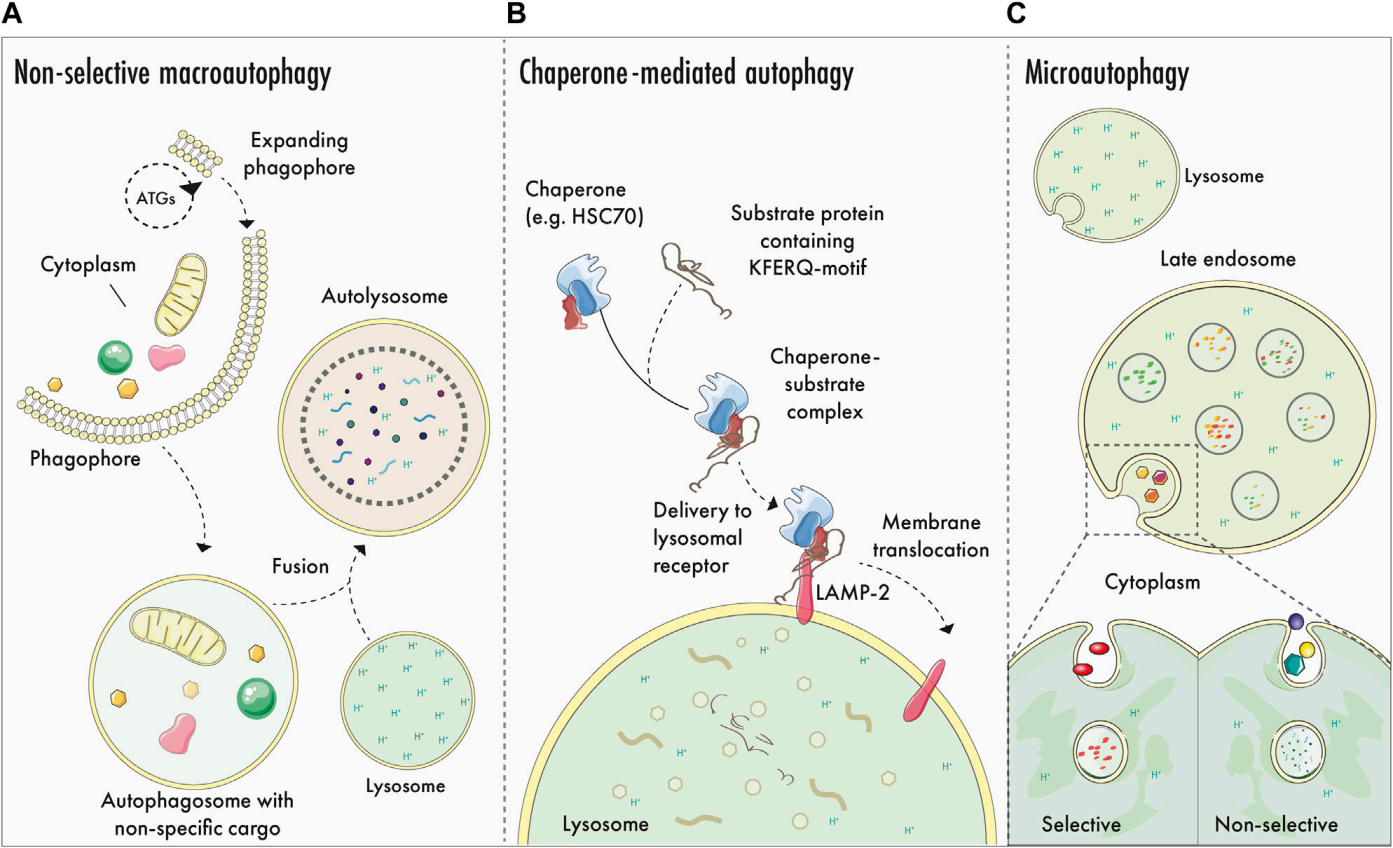
Generalised autophagy logistics: macroautophagy, CMA and microautophagy. **(A)**–Non-selective macroautophagy: ATG proteins participate in phagophore biogenesis and expansion, generating a double membrane structure that encapsulates portions of cytoplasmic matter. Leading edges of the expanding phagophore undergo abscission to form the autophagosome, which matures and eventually fuses with an acidic endolysosome to yield an autolysosome. Cargo is degraded by hydrolytic enzymes originating from the lysosome and is recycled to fuel cellular anabolism and signalling. **(B)**–Chaperone mediated autophagy (CMA): CMA involves the selective sequestration of proteins containing a KFERQ-motif. Here, cytosolic proteins are recogniced by chaperones that mediate the transportation to the lysosomal membrane, where the transmembrane receptor LAMP-2 binds to and translocates the cargo into the lysosomal lumen for elimination. **(C)**–Selective and non-selective microautophagy. Cytosolic cargo is degraded selectively or non-selectively via lysosomal or late endosomal membrane invagination and internalisation. Once internalised, lysosomal hydrolytic enzymes degrade the cargo.

Our cells have evolved several mechanisms to offset the cumulative molecular effects of a lifetime of exposure to stress and damage (Figure 1). Additionally, cells and tissues must maintain a balance between building and burning i.e. anabolism and catabolism. Autophagy (meaning “*to eat oneself*”) is a dynamic mechanism that ensures this balance, where cells degrade cellular components in acidic endolysosomes (Eskelinen, 2019; Suomi and McWilliams 2019; Collier et al., 2021a; Klionsky et al., 2021b). In a general sense, autophagy fulfills several critical functions: first, it prevents the age-dependent accumulation of harmful cellular components e.g., dysfunctional organelles, proteinaceous aggregates, and other components. Secondly, autophagy is a recycling pathway, and the degradation of cargo results in the release of macromolecules that feed anabolic pathways. Third, it has emerged that not all steps and components of the autophagy pathway are destined for degradation—autophagy has an important signalling function in cells, which contributes to immune and vesicle signalling pathways (Collier et al., 2021a). Autophagy likely evolved to sustain cells during prolonged instances of nutrient deprivation or starvation. Accordingly, autophagy has emerged as an important mechanism that underpins both animal development (Allen and Baehrecke, 2020) and longevity, because caloric restriction in several organisms has been linked to lifespan extension (de Cabo and Mattson 2019). Autophagy is a popular topic in fitness and health culture, and several compounds are promoted to ‘boost’ or ‘enhance’ autophagy–despite the absence of a robust clinical-grade assay that can repeatedly measure longitudinal autophagic activity in humans. The contribution of tissue-specific macroautophagy enhancement to mammalian healthspan remains to be determined.

A common assumption is that physiological autophagy is a dormant process that needs to be ‘activated’ in some way (either by prolonged starvation, or pharmacological stimulus). It is difficult to attribute the origin of this popular assertion, but we speculate it may stem from critical differences in the levels of basal autophagy in adherent cell culture systems compared to turnover in complex, vascularised tissues. In most tissue culture systems, adherent cells receive a relatively constant supply of ample nutrients (amino acids, lipids and often saturating glucose levels). Accordingly, the abundance of nutrients means that cultured cells have minimal requirements for self-catabolism, and levels of steady-state autophagy remain relatively low. Accordingly, the study of autophagy under laboratory conditions requires its induction or activation (above baseline levels) through straightforward deprivation paradigms or pharmacological means (e.g., starvation or mTORC1 inhibition) (Klionsky et al., 2021a). Such treatments induce a robust and often rapid response of sufficient magnitude, whereby each step can be controlled and studied at high resolution using a variety of methods (Klionsky et al., 2021a). These paradigms have been exploited widely over the last 3 decades and have been invaluable in deciphering the mechanistic modulation of macroautophagic flux.

The roots of autophagy research predate induction protocols and involved the characterisation of autophagic structures at physiological steady state in mammalian tissue preparations (in both tissue homogenates and in tissue sections) (Clark 1957; Ashford and Porter 1962; De Duve 1963; Arstila and Trump 1968; Ohsumi 2014). Decades later, pioneering studies of autophagy reporter mice (LC3-GFP animals, and subsequent ratiometric reporters) revealed the presence of constitutive autophagy events within tissues *in vivo* (Mizushima et al., 2004; Kuma et al., 2017). Indeed, nutrient restriction can augment or enhance autophagic activity in some tissues (Mizushima et al., 2004; Tanida et al., 2005; Wong et al., 2013). It remains to be determined if all non-endolysosomal LC3 puncta are entirely involved in degredation, or if a proportion of these could reflect some other non-degredative signalling events *in vivo*.

Starvation-induced autophagy in both cells and tissues induces the non-selective or bulk/general elimination of cytoplasmic components. In general, although mitochondria can be eliminated by starvation-induced autophagy, they do not appear to be a preferential target *in vivo*. Parallel studies of mitophagy and macroautophagy reporter mice revealed that overnight starvation induces an elevated level of mitophagy *in vivo*, but levels of macroautophagy are more significantly pronounced (McWilliams et al., 2018a). Interestingly, prolonged fasting augments piecemeal mitophagy in zebrafish tissues, and this response appears to be developmentally-dependent (Wrighton et al., 2021). To this effect, mitochondria are particularly vulnerable during starvation-induced macroautophagy. Altered nutrient status triggers several adaptive mechanisms, such as altered mitochondrial network dynamics (Rambold et al., 2011) and triggers DGAT1-dependent lipid droplet (LD) biosynthesis to offset mitochondrial dysfunction induced by long chain carnitine species (Nguyen et al., 2017).

Autophagy-related (ATG) genes encode enzymes that operate in complexes to regulate several steps of the autophagy pathway, including phagophore biogenesis and dynamics (Dikic and Elazar, 2018). The phagophore is a double membrane-bound structure comprised of two leading edges, which advance to engulf cargo. The leading edges of the phagophore seal by abscission to form the autophagosome (Melia et al., 2020). Numerous membrane sources have been implicated in autophagosome biogenesis, and this topic has been reviewed elsewhere (Gómez-Sánchez et al., 2021). The recently reported ‘hybrid pre-autophagosomal structure (HyPAS)’ is noteworthy, as it may unify the ER-centric and endosome-centric schools of thought on membrane sources (Kumar et al., 2021a).

Autophagosomes are transient organelles that contain sequestered cargo and ultimately fuse with acidic subcompartments of the endolysosomal pathway; lysosomes or late endosomes (Walker and Ktistakis, 2020). The fusion of autophagosomes and lysosomes generates autolysosomes, characterised by the loss of the inner autophagosomal membrane. Here, sequestered cargo is degraded by lysosomal hydrolases, and macromolecules are released into the cytosol for recycling (Suomi and McWilliams 2019). As the process of autophagy terminates, the lysosomal pool is regenerated by the process of autophagosome-lysosome reformation (ALR) (Yu et al., 2010) thereby enabling cycles of autophagy to continue. From a big picture perspective, ‘Atg8ylation’ was recently proposed as a term to describe the conjugation of ATG8 proteins to membranes, akin to protein ubiquitylation. This revised paradigm could capture many canonical and non-canonical manifestations of autophagy signalling within a homeostatic continuum of ‘general stress and membrane remodelling’ responses—reviewed in (Deretic and Lazarou, 2022; Kumar et al., 2021b).

Autophagy proteins also participate in a wide range of non-autophagy-associated processes (Galluzzi and Green 2019). Studies in *Atg7*-null mouse fibroblasts have shown the involvement of *Atg7* in repressing pro-apoptotic events through its interaction with p53 during nutrient deprived conditions, and by repressing the pro-apoptotic properties of caspase 9 (Han et al., 2014; Collier et al., 2021b). The activities of *Atg7* influence skeletal muscle development, pancreatic exocrine and endocrine capacity and underpin neuronal survival and integrity neuronal survival and integrity (reviewed in Collier et al., 2021a). Autophagy is particularly important for the homeostatic maintenance and integrity of long-lived post-mitotic cells, such as neurons (Suomi and McWilliams, 2019). Initial mouse studies demonstrated perinatal lethality in *Atg5-* and *Atg7*-null mice (Kuma et al., 2004; Komatsu et al., 2005), thus it was initially difficult to ascertain the physiological significance of autophagy in the nervous system. However, studies of conditional mouse models with selective loss of neuronal autophagy revealed authentic neurodegenerative phenotypes, thereby confirming the preclinical significance of autophagy for the nervous system (Hara et al., 2006; Komatsu et al., 2006). Strikingly, neural-selective restoration of *Atg5* expression in whole-body *Atg5-*null mice rescues the lethality phenotype (Yoshii et al., 2016). Although the animals still harbour a range of tissue impairments, this elegant study demonstrated the impact and importance of neural autophagy for systemic physiology (Yoshii et al., 2016).

A logical assumption arising from *Atg*-deficient mice is that human embryos with similar inactivating mutations would be inviable. Strikingly, several clinical cases of ‘congenital autophagy deficiency’ have recently been described. These pathogenic variant mutations in *ATG7* and *ATG5* do not cause human perinatal lethality, yet they have detrimental consequences for neurodevelopment, with patients manifesting both neuropathology and endocrine dysfunction (Kim et al., 2016; Collier et al., 2021b; Baselli et al., 2022). Together these mouse and human studies firmly demonstrate that autophagy is a disease-relevant process of outstanding therapeutic importance for neurological and metabolic indications.

## How recycling mitigates mitochondrial meltdown: The role of selective autophagy

Starvation promotes the non-selective breakdown of cellular matter; however, cells also exploit autophagic degradation during nutrient replete or steady-state conditions. Selective autophagy pathways have evolved to enable adaptation to changing metabolic demands, as well as to control cellular organelle quality and abundance (Kirkin and Rogov 2019; Lamark and Johansen, 2021). Targeted or selective degradation is a feature of numerous autophagy pathways, including chaperone mediated autophagy (CMA) and microautophagy (reviewed in Kaushik and Cuervo 2018 and Schuck 2020, respectively). Selective autophagy is a term that generally refers to pathways that promote the targeted elimination of organelles and their subcompartments *via* selective autophagy receptors (SARs) (Kirkin and Rogov 2019).

The best studied selective autophagy pathway is mitophagy: which promotes mitochondrial turnover (Killackey et al., 2020; Montava-Garriga and Ganley 2020; Zachari and Ktistakis 2020; Ktistakis 2021). The first known conceptual reference to mitochondrial elimination in the literature dates back to the prodigious work of Margaret and Warren Lewis in 1915, who observed ‘degenerating mitochondria’ in some of the earliest mammalian cell culture experiments (Lewis and Lewis, 1915). Pioneering studies using transmission electron microscopy (TEM) revealed instances of encapsulated mitochondria within lysosomes and other degradative compartments (Clark 1957; Ashford and Porter 1962; Novikoff and Essner 1962). The present-day interest in mitophagy has arisen from its tantalising links to neurodegenerative disease pathology, most notably to rare familial Parkinson’s disease (PD) (McWilliams and Muqit 2017) and Alzheimer’s disease (Fang et al., 2019). The causes of idiopathic PD remain unclear, thus studying rare genetic forms of Parkinsonism has provided important insights on possible therapeutic targets, which may have broader relevance.

**FIGURE 2:**
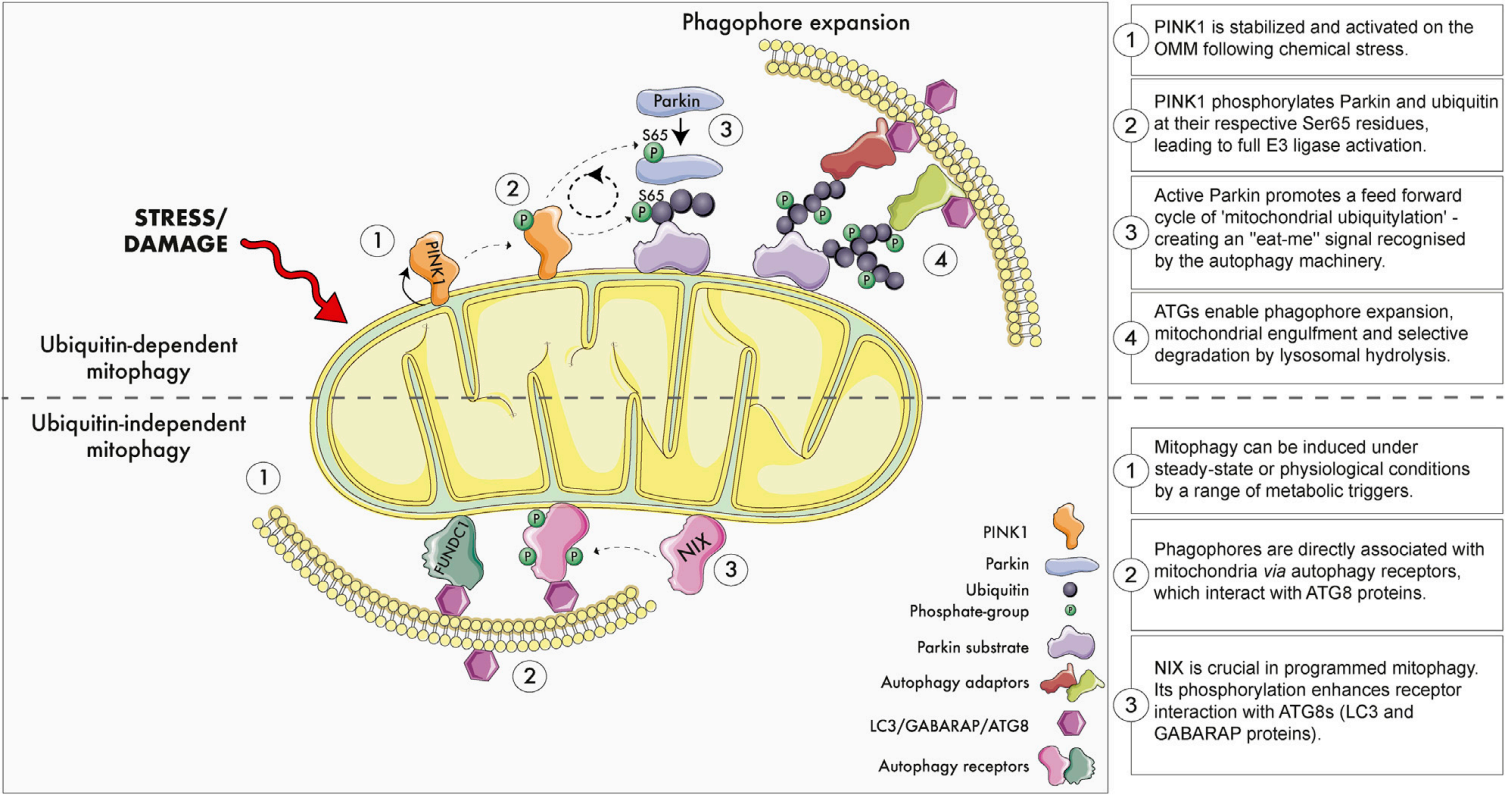
Ubiquitin dependent and independent mitophagy. Upper panel: The best studied ubiquitin-dependent mitophagy mechanism is the PINK1-Parkin-dependent pathway. Briefly, extreme chemical stress promotes mitochondrial damage which triggers PINK1 stabilisation and activation on the outer mitochondrial membrane (OMM). PINK1 recruits ubiquitin and the E3 ligase Parkin to the mitochondria and phosphorylates their respective Ser65 residues. Feed-forward amplification cycle signals result in coating of damaged mitochondria with ubiquitin, which promotes the recruitment of autophagy adaptors, phagophore biogenesis and engulfment. Engulfed mitochondria within autophagosomes are destroyed upon fusion with acidic endolysosomal subcompartment. Although PINK1 and Parkin promote mitochondrial clearance in cultured proliferating cells, several studies demonstrate this pathway does not regulate physiological mitophagy within tissues. Lower panel: Ubiquitin-independent mitophagy. Basal and programmed mitophagy occur in tissues, likely in response to altered metabolic demands, states of insults that damage mitochondria. Phagophores form on mitochondria through the interaction of selective autophagy receptors (e.g., NIX, FUNDC1) with ATG8 proteins. NIX/BNIP3L is especially important during programmed mitophagy and metabolic maturation. NIX/BNIP3L phosphorylation enhances its association with ATG8 proteins. Loss of NIX/BNIP3L alters physiological mitophagy in the retina and reticulocytes. Several of the most promising therapeutic strategies to modulate mitophagy operate via ubiquitin-independent mechanisms.

Emerging evidence suggests several molecular pathways control the autophagic elimination of mitochondria in distinct physiological contexts. Broadly speaking, these mitophagy pathways can be classified as either ubiquitin-dependent or independent (Figure 2) (Khaminets et al., 2016). One of the most studied paradigms is PINK1-Parkin dependent mitophagy as these genes encode a ubiquitin kinase (PINK1; *PARK6*) and RBR E3 ubiquitin ligase (Parkin; *PARK2*) that are mutated in rare forms of early onset PD (McWilliams and Muqit 2017; Whitworth and Pallanck, 2017). Under highly specific conditions where mitochondrial membrane potential is abolished in cultured cells, PINK1 becomes stabilised and activated on the outer mitochondrial membrane (OMM) (Narendra et al., 2008; Narendra et al., 2010). Active PINK1 phosphorylates both Parkin and ubiquitin at their respective Ser65 residues, triggering feedforward amplification cycles of ‘mitochondrial ubiquitylation’ (Bayne and Trempe, 2019; Ordureau et al., 2018; Dunkerley et al., 2022; Goodall et al., 2022). Here, damaged mitochondria are decorated with chains of phospho-ubiquitin, which serves as an “*eat-me*” signal that is recognised by the autophagic machinery. This ultimately leads to the selective elimination of the damaged organelle.

Recent work has shown that physiological mitophagy readily occurs across all tissues in the absence of a functional PINK1-Parkin *in vivo* (McWilliams et al., 2018a; 2018b; Lee et al., 2018; Wrighton et al., 2021) but also in cultured human neurons *in vitro* (Ordureau et al., 2021). This is not surprising because animal models and patients lacking PINK1 or Parkin do not manifest overt mitochondrial phenotypes, or a build-up of damaged mitochondria within tissues (Goldberg et al., 2003; Perez and Palmiter 2005; Yang et al., 2019; Wrighton et al., 2021).

Myriad differences exist between proliferating HeLa cells and post-mitotic neurons, yet endogenous PINK1 activation robustly promotes Parkin-dependent mitochondrial ubiquitylation in mature primary mouse neurons (McWilliams et al., 2018a) and human iNeuron cultures (Ordureau et al., 2020). Although the PINK1/Parkin pathway is dispensable for physiological mitophagy, it is important to highlight that the molecular findings of the past decade have elaborated a signalling pathway of clinical importance. Namely, loss of PINK1-dependent phosphorylation of Parkin^Ser65^ leads to human Parkinsonism (McWilliams et al., 2018b). Still, basal mitophagy is unaffected in both mice and humans with inactivating mutations at Parkin^Ser65^ (McWilliams et al., 2018a). Consistent with this, the mitophagy-independent functions of PINK1-Parkin have gained increasing physiological and pre-clinical relevance. Notably, impaired PINK1-Parkin signalling leads to infection-associated autoimmune pathology which promotes authentic nigrostriatal pathology (Matheoud et al., 2019). Hence, the PINK1/Parkin-pathway has clear clinical relevance, yet its physiological significance appears more convincing for neuroimmune homeostasis, rather than mitophagy as initially described. It will be exciting to determine how the therapeutic reactivation of this pathway impacts neuroimmune integrity. The mitophagy-independent functions of PINK1/Parkin signalling are now increasingly recognised, and have been reviewed elsewhere (Pollock et al., 2021; Lizama and Chu, 2021; Villa et al., 2018; Chen et al., 2022). The recent profiling of ubiquitylated mitochondrial substrates common to both mouse and human neurons will prove informative to further define the physiological, mitophagy-independent functions of Parkin (Antico et al., 2021).

Intriguingly, the most recent structural studies of human PINK1 suggest its activation mechanism may involve direct regulation by mitochondrial reactive oxygen species (mtROS) (Gan et al., 2022). The generic term ‘ROS’ encompasses a broad selection of radical and non-radical O_2_ species (Murphy et al., 2022), and a vast body of literature demonstrates supraphysiological ROS generation promotes cell dysfunction (Lennicke and Cochemé, 2021), also reported for cells and tissues with dysfunctional PINK1/Parkin signalling (Hoepken et al., 2007; Pridgeon et al., 2007; Anichtchik et al., 2008; Narendra et al., 2010; Priyadarshini *et* al. 2013). However, ROS are also potent metabolic signalling molecules that play pivotal roles in physiology (Finkel, 1998; Schieber and Chandel, 2014; Sies and Jones, 2020; Sies et al., 2022), particularly in the nervous system where they exert cell-type specific regulation (Baxter and Hardingham, 2016; Vicente-Gutierrez et al., 2019). Some hypothesise that ROS overproduction may confer a beneficial hormetic effect that facilitates cellular adaptation to stress (mitohormesis) (Ristow and Schmeisser, 2014). It is tempting to speculate loss of PINK1-dependent Parkin activation disrupts an age-related hormetic adaptation to mtROS, leading to the early degeneration of long-lived neurons. Resolving this emerging interplay between immunity, mitochondrial signalling and redox metabolism may finally triangulate the pathomechanism of PINK1/Parkin-associated Parkinsonism.

## Emerging concepts in physiological mitophagy

Targeting physiological mitophagy has major translational relevance for the nervous system and beyond. Takahashi and colleagues recently described a modified PROTAC (mito-AUTAC) that can be used to selectively eliminate mitochondrial fragments by mitophagy. Importantly, mito-AUTAC promotes mitophagy by a PINK1/Parkin-independent mechanism (Takahashi et al., 2019). The successful autophagic clearance of mutant huntingtin was also accomplished using a similar approach (Li et al., 2019). A ‘ubiquitin-bypass’ approach was also reported to induce mitophagy, through ectopic tethering of ULK1 or using a FIP200 peptide (Vargas et al., 2019). These innovative approaches will surely advance therapeutic efforts to target mitophagy, with broader relevance for other selective autophagy pathways.

In cultured cells, PINK1-Parkin-independent mitophagy can be induced using therapeutic iron chelators or hypoxia (Zhang et al., 2008; Allen et al., 2013; Montava-Garriga and Ganley 2020). HMG-CoA reductase inhibition has also been reported to alter mitophagy (Andres et al., 2014). The therapeutic molecule ivermectin was also shown to induce PINK1-Parkin-independent mitochondrial turnover, and this paradigm revealed critical contributions of the endoplasmic reticulum as a platform for mitophagosome biogenesis, that regulates the dynamics of ubiquitin-dependent mitophagy (Zachari et al., 2019). Ubiquitin-independent mitophagy depends on the recruitment and binding of the phagophore directly to specific OMM proteins that serve as mitophagy receptors, triggering phagophore nucleation, expansion and engulfment (Montava-Garriga and Ganley 2020). For example, the phosphorylation of the ubiquitin-independent receptor NIX/BNIP3L increases its engagement with ATG8 proteins LC3/GABARAP, promoting mitochondrial encapsulation and elimination (Schweers et al., 2007; Rogov et al., 2017).

Importantly, familial PD continues to provide surprises in the mitophagy field: recent work has identified the PD-related kinase LRRK2 as a *bona fide* regulator of physiological mitophagy in mammals, with increased kinase activity leading to diminished levels of mitophagy, and loss of LRRK2 triggering enhanced mitophagy *in vivo* (Singh F. et al., 2021). Excitingly, LRRK2 is a therapeutic target with several advanced modalities, including small molecule and antisense therapies in clinical trials (Tolosa et al., 2020; Singh and Ganley 2021). The mitochondrial disease associated FBXL4, was also recently implicated in the regulation of mammalian mitophagy (Alsina *at al.* 2020).

Aside from quality control, mitophagy may play an important role in metabolic plasticity, by fine tuning mitochondrial number and composition to basal metabolic demand. This is particularly important in tissue development and metabolic maturation. “Programmed mitophagy” enables the cellular metabolic switch from oxidative phosphorylation towards increased glycolysis, also in aerobic conditions. This process has been described to occur in cancer cells, however, it is also an important feature of tissue development in the retina and ocular tissues (Esteban-Martínez et al., 2017; McWilliams et al., 2019; and reviewed in Morishita, 2022) and hematopoietic system (Aerbajinai et al., 2003). Notably, the late embryonic heart and kidneys also exhibit waves of mitophagy that occur within restricted developmental windows, thought to be important for perinatal metabolic maturation (McWilliams et al., 2016).

Importantly, mitophagy does not proceed in isolation and can synergise with other pathways to safeguard metabolic homeostasis and cellular survival. Recent work demonstrates that iron chelation induces both mitophagy but also DGAT1-dependent lipid droplet (LD) biosynthesis. Here, LDs appear to neutralise lipotoxicity arising from iron depletion, which would otherwise impair lysosomal activity. Interestingly, loss of DGAT1 in *Drosophila* (*via* tissue-specific depletion of the DGAT orthologue *mdy*) impairs basal mitophagy in the brain and promotes locomotor dysfunction (Liesa 2022; Long et al., 2022; Long and McWilliams 2022). This suggests that targeting mitophagy in disease might be accomplished by unconventional or lateral strategies. In agreement with this, genetic inhibition of DGAT1 in mice leads to lysosomal defects (Hung and Buhman 2019), and the combined genetic ablation of DGAT1 and DGAT2 leads to the accumulation of damaged mitochondria in adipocytes (Harris et al., 2011).

## How to know what happens *in vivo*: Monitoring mitophagy in tissues

Mitophagy has been studied extensively *in vitro,* and this work has been instrumental in identifying novel regulatory features of the pathway at high mechanistic resolution. However, it is essential to understand how mitophagy proceeds within complex tissue systems, as the dynamics of organelle biology may differ *in vivo*. Like macroautophagy, selective mitochondrial turnover has also emerged as a basal cellular process occurring independently of mitochondrial damage or cellular stress to maintain mitochondrial network functionality and adaptability to different metabolic needs of the cell (Palikaras et al., 2018; Suomi and McWilliams 2019; Killackey et al., 2020; Montava-Garriga and Ganley 2020).

However, the monitoring of mitochondrial turnover *in vivo* is still a relatively recent advance. The generation of mitophagy reporter mice has revolutionised this field of research, enabling further understanding of selective mitochondrial destruction in physiological conditions (Kuma et al., 2017). Most mitophagy reporters rely on pH-dependent assays enabled by fluorescent proteins with different physicochemical properties. A well-established and extensively validated model is the *mito*-QC reporter mouse, which expresses a mCherry-GFP tandem reporter on the OMM via the mitochondrial targeting sequence of FIS1 (FIS1mt101-152) (McWilliams et al., 2016). *mito*-QC is ubiquitously expressed across cell- and tissue-types, illuminating mitochondria in green and red (yellow) during physiological conditions. Upon the delivery of encapsulated mitochondria to the lysosome, the acid-labile GFP signal is quenched while mCherry fluorescence remains stable. Thus, mCherry-only positive puncta (mitolysosomes) can be quantified as a readout of mitophagy (McWilliams et al., 2016; McWillams and Ganley 2019). mt-Keima is another mitophagy reporter mouse which reporter is localised to the mitochondrial inner matrix cytochrome *c* oxidase subunit 8 (COXVIII), expressed from the *Hipp11* locus in FVB mice (Sun et al., 2015). This system enables mitophagy detection through the excitation wavelength shift upon mitolysosome formation. Importantly, both reporter mouse models establish that mitophagy occurs at steady state levels within tissues (Sun et al., 2015; McWilliams et al., 2016). A matrix-tagged variant of *mito*-QC has also been reported, known as *matrix*-QC (Lee et al., 2018; Long et al., 2022), and shows identical patterns to *mito*-QC. Elegant work by the Goessling laboratory systematically compared mCherry-GFP and mt-Keima mitophagy biosensors *in vivo*, demonstrating they both report physiological PINK1/Parkin-independent mitophagy in zebrafish tissues (Wrighton et al., 2021). A tandem-tag reporter with an OMP25 mitochondrial targeting sequence also demonstrates equivalent patterns (Yamashita et al., 2021), and MitoTimer is also used to study mitochondrial homeostasis *in vivo* (Laker et al., 2014).

Reporter mice for other autophagy forms have been generated (reviewed in Kuma et al., 2017), such as the GFP-LC3 macroautophagy reporter mouse (Mizushima et al., 2004), and tandem-tag autophagy reporter GFP-mCherry-LC3 (also referred to as the *auto*-QC mouse–*Rosa26* expression of mCherry-GFP-Map1lc3b; McWilliams et al., 2018a; Singh F. et al., 2021). The CMA reporter mouse is a tool for *in vivo* monitoring of the selective degradation pathway of proteins containing a KFERQ-motif *via* chaperone mediated autophagy (Dong et al., 2020). These reporter models have enhanced our understanding of autophagic events *in vivo*, illuminating important differences between physiological mitophagy from the stress-responses initially characterised under *in vitro* conditions. The development of *in vivo* reporters for other selective autophagy pathways, including pexophagy, lipophagy and ribophagy will be crucial to understand how these degrative and non-degradative autophagy signalling contributes to cellular homeostasis in health and disease.

## Focus on mitophagy in aging

Vertebrate autophagy deficiency leads to reduced lifespan, progeria and neuropathology (Kuma et al., 2004; Simonsen et al., 2008; Yoshii et al., 2016; Collier et al., 2021b). Understanding the age-dependent regulation of macroautophagy and selective autophagy pathways may reveal much needed therapeutic targets for age-related pathology (Aman et al., 2021; Kaushik et al., 2021). Much of our current framework of autophagy in aging is derived largely from studies on cultured cells and short-lived model organisms, which suggests that autophagic activity progressively declines as a function of age. Data for CMA are becoming clearer, with an initial decrease observed in (Cuervo and Dice, 2000).

Age-dependent decreases in autophagy have been reported in budding yeast (*Saccharomyces cerevisiae*) as a result of decreased vacuolar pH resulting in reduced lifespan and compromised mitochondrial function (Hughes and Gottschling 2012). Similarly, age-dependent decreases in mRNA levels of Atg2, Atg8a and Atg18 were also previously reported in *Drosophila* with *Atg8a*- flies showing increased formation of CNS ubiquitin^+^ protein inclusions compared to age-matched controls (Simonsen et al., 2008). Enhanced expression of neuronal Atg8 promotes *Drosophila* longevity, as the lifespan of *APPL-Gal4/EP-UAS-Atg8a* flies exceeds their wild type counterparts by 56% (maximal lifespan of these flies is extended to 96 days compared to 88 days for wild type). (Simonsen et al., 2008). Nematodes are a powerful system to understand the regulation of autophagy in aging and longevity (Hansen et al., 2008; Konstantinidis and Tavernarakis, 2021). Here, autophagy is regulated in a tissue-specific fashion and reportedly declines with age (Chang et al., 2017), yet there may be cell-type and tissue-specific exceptions to this (Chapin et al., 2015). A comparative analysis of neural aging and macroautophagy across model systems is discussed in (Kallergi and Nikoletopoulou, 2021). Mitophagy is also reported to decline with age in nematodes (Palikaras et al., 2015) and flies (Schmid et al., 2022). The reciprocal expression of negative regulators may also explain declining autophagy levels observed in aging tissues. Rubicon is reported to negatively regulate autophagy and its enhanced expression is observed in aged tissues from nematodes, flies and mice (Nakamura et al., 2019). Loss of Rubicon extended lifespan in *C. elegans* and delayed the onset of age-associated hepatic and renal fibrosis in mice, in addition to lewy-like striatal pathology (Nakamura et al., 2019). Haploinsufficiency of the pro-autophagic protein Ambra1 was recently shown to accelerate age-related pathology in the retina, a CNS tissue of high metabolic demand (Ramírez-Pardo et al., 2022). As a form of selective autophagy that targets specific proteins, CMA is already advanced as a therapeutic target with proof-of-concept. Age-related decreases in CMA were initially reported from the analysis of rat tissues (Cuervo and Dice, 2000) and most recently from AD patient data *via* a CMA activation score (Bourdenx et al., 2021). Autophagy-enhancing strategies for longevity have been previously reviewed (Hansen et al., 2018).

Regarding the longitudinal study of mitophagy during mammalian brain aging, little data currently exist because reporter mice are a rather recent advance in the field. Furthermore, mammalian aging studies are not always feasible, and may be limited in scope, due to different regulations pertaining to animal research. Mitophagy has been profiled at a regional level, for example in dentate gyrus (DG) of young and old mt-Keima mice, with reduced fluorescence observed in 21-month-old animals (Sun et al., 2015). This observation has contributed to the idea that mitophagy declines with aging. Yet it is difficult to exclude that the physicochemical properties of the mt-Keima reporter may hinder precise quantitation of mitophagy at the single-cell level. Importantly, whilst FVB mice are useful for transgenesis, two decades of phenotypic and pre-clinical profiling have described a plethora of neurological and behavioural abnormalities, with neurodegeneration, blindness (Chang et al., 2002; Mineur and Crusio 2002; Pugh et al., 2004; Farley et al., 2011; Eltokhi et al., 2020; Ganley et al., 2021) and altered mitochondrial homeostasis (Singh S. et al., 2021). Hence, it is difficult to dissociate whether the loss of fluorescence observed in these animals is a function of aging, or from natural pathology characteristic to FVB mice. Studies using additional reporter mice genetic backgrounds with a natural aging phenotype will be necessary to clarify this further.

Evolutionary selection pressures for short- and long-lived model organisms are distinct. Thus, would we expect an age-dependent decline in mitophagy to be a general feature of the mammalian nervous system? With an enormous degree of heterogeneity in cellular subtypes, it would be remarkable if mitophagy was regulated in the same way across years and decades of life. Regardless, the notion of age-dependent decreases in mitophagy is a captivating idea. A natural corollary suggests that the targeted enhancement of mitophagy might offset progressive neurodegeneration and promote longevity. Yet recent observations suggest a general decrease in mitophagy may not be a clear-cut hallmark of natural brain aging in mammals.

For example, increased autophagic activity characterises age-associated ocular hypertension in pre-clinical rodent models and patient specimens (Nettesheim et al., 2020). Interestingly, LC3-II levels in the long-lived rodent naked mole rat (NMR) remain unchanged during aging, suggesting that autophagy levels remain stable throughout time (Triplett et al., 2015). As the longest-lived rodent, the NMR is especially interesting for aging studies given their stable genomic integrity, cancer resistance and robust proteostasis throughout their lifespan. However, measurements of LC3-II need to be interpreted with caution, as their method of normalisation can result in very different interpretations, given that LC3 levels vary between cells, tissues and individuals (Klionsky et al., 2021a). Changes in LC3 alone do not reflect changes in mitophagy, which require the use of reporter systems to distinguish selective turnover from non-selective macroautophagy (McWilliams and Ganley, 2019).

Hou et al., 2018 quantified phosphorylated poly-ubiquitin chains (p-S65-Ub) in human post-mortem brain specimens of healthy aged and Lewy body disorder (LBD) patients. The authors analysed PD-relevant brain regions including the substantia nigra (SN), hippocampus, amygdala, putamen and nucleus basalis of Meynert (nbM). Phospho-ubiquitin levels were found to increase in an age-dependent manner in the amygdala and nbM of healthy aging patients, while SN levels remained disease-dependent. Hippocampal p-S65-Ub levels were more strongly associated with pathology, however a significant age-dependent increase was also observed in this brain region. These data suggest that mitophagy may be modified by aging. However, loss of Parkin S65 phosphorylation promotes locomotor dysfunction in Parkin^S65A/S65A^ mice and PD in humans, without any changes in basal mitophagy (McWilliams et al., 2018a). Thus, quantification of pSer65-Ub levels relative to total ubiquitin levels would prove extremely informative, given that PINK1 remains the only identified ubiquitin kinase to date. Elevated p-S65-Ub levels in post-mortem brains of both healthy aged and LBD patients may reflect increased mitophagy during aging and pathology, possibly as a mechanism for maintaining cellular and tissue homeostasis. This *in vivo* human data provides an interesting counter argument to the notion that mitophagy decreases through aging and disease. It will be exciting to determine if ‘mitochondrial ubiquitylation’ always leads to lysosomal degradation, or if this modification has an uncharacterised signalling function i.e., does phospho-ubiquitin always represent a mitochondrial “eat-me” signal, or might it act as some other adaptive signal in particular physiological contexts?

Regardless of whatever mitophagy or macroautophagy pathways being driven or diminished, successful turnover depends upon lysosomal efficiency which is critical for neural integrity (Ballabio and Bonifacino, 2020; Nixon, 2020; Lee et al., 2022; Udayar et al., 2022). The abundance, position, activity, pH and composition of lysosomes are parameters that are crucial for mitophagy. A thorough discussion of lysosomal homeostasis in the aging nervous system is beyond the scope of this review, but this topic has been extensively reviewed elsewhere (Nixon, 2020).

## Conclusions and future perspectives

Living longer is changing our global population, with major ramifications for brain health and cognition (Nagaratnam et al., 2022). Why some humans experience accelerated neural aging compared to others remains to be fully understood (Gonzales et al., 2022). An assortment of interrelated life factors impact age-related brain decline in humans (e.g., not limited to - congenital factors, early-life development, socioeconomic disparities, wellbeing, access to healthcare, metabolic health, circadian rhythm, and environmental enrichment) (Chan et al., 2018; Smith et al., 2020; Vidal-Pineiro et al., 2021). Accordingly, no single experimental system can adequately capture the full repertoire of influences that promote or prevent neural aging.

Autophagy and mitophagy are pathways of outstanding clinical interest with major relevance for neural integrity because, human neural function depends upon quality control over a timespan of decades. Although mitophagy levels decline in short-lived model organisms, it remains unclear if decreased levels of mitophagy are a hallmark of all cell types during natural aging in the mammalian nervous system. Furthermore, it remains unclear if certain cerebral regions and cellular subtypes exhibit greater susceptibility to age-related changes than others. For example, cortical thickness is a widely used metric in human aging studies (Frangou et al., 2022) but the first large-scale heterochronic datasets (brain charts) are only beginning to emerge (Bethlehem et al., 2022). Delineating the regional and cell subset-specific regulation of mitophagy will be critical to develop neuroprotective interventions that might improve healthspan or even reverse human age-related degeneration.

It will also be exciting to discover the possible interplay between emerging mitophagy pathways, and age-dependent pathology in the mammalian nervous system. Crosstalk between basal mitophagy and other mitochondrial responses e.g., outer membrane remodeling in response to infection (Li et al., 2022) and signalling or degradative mitochondria-derived vesicle (MDV) formation also represent intriguing avenues for future investigation. Whether elevated levels of mitophagy are beneficial for neural integrity also remains a mystery. What is the “minimum effective threshold” of basal mitophagy or macroautophagy required to safeguard neural integrity? How can we control or fine tune mitophagy to prevent a deleterious outcome? What is the relationship between physiological mitophagy and contemporary concepts in geroscience, such as epigenetic aging (Horvath et al., 2016; Lu et al., 2017)? The continued development and characterisation of novel tools presents a unique opportunity to resolve these longstanding questions.

What is the role of selective autophagy in the neuroprotective effects afforded by behavioral interventions? There are clear pro-longevity effects of exercise for cognitive, cerebrovascular and systemic health (Oudbier et al., 2022). Indeed, autophagy and mitophagy are impacted by exercise in different model systems (He et al., 2012)*.* Developing robust protocols and pharmacological strategies to augment selective autophagy pathways in humans represents a major challenge, because we do not have rapid, non-invasive assays that can reliably monitor distinct forms of autophagy in the clinic (at point of care). Equally, such clinical assays would need to distinguish between the degradative and signalling functions of autophagy (not a trivial task, even under laboratory conditions). Moreover, it remains unknown if changes in serum levels of autophagy markers reflect alterations in cell or tissue-specific autophagy pathways. These are challenging questions, but also exciting opportunities that will lead to a better understanding of physiological mitophagy in tissue development, disease and repair.
